# An Apparent Gas Permeability Model for Real Gas Flow in Fractured Porous Media with Roughened Surfaces

**DOI:** 10.3390/polym13121937

**Published:** 2021-06-10

**Authors:** Tao Wu, Qian Wang, Shifang Wang

**Affiliations:** 1 Hubei Key Laboratory of Optical Information and Pattern Recognition, School of Mathematics and Physics, Wuhan Institute of Technology, Wuhan 430205, China; wutao@wit.edu.cn (T.W.); wtlaser@126.com (Q.W.); 2 School of Physics and Mechanical & Electrical Engineering, Hubei University of Education, Wuhan 430205, China; 3 Institute of Theoretical Physics, Hubei University of Education, Wuhan 430205, China

**Keywords:** fractured porous media, apparent gas permeability, fractal theory, roughened surfaces

## Abstract

The investigation of gas transport in fractured porous media is essential in most petroleum and chemical engineering. In this paper, an apparent gas permeability model for real gas flow in fractured porous media is derived with adequate consideration of real gas effect, the roughness of fracture surface, and Knudsen diffusion based on the fractal theory. The fractal apparent gas permeability model is obtained to be a function of micro-structural parameters of fractured porous media, relative roughness, the pressure, the temperature, and the properties of gas. The predictions from the apparent gas permeability model based on the fractal theory match well with the published permeability model and experimental data, which verifies the rationality of the present fractal apparent gas permeability model.

## 1. Introduction

The behavior of gas transport in porous media has received increased critical attention in recent decades due to many engineering applications such as the development of shale gas reservoir, water purification, industrial filtration, synthesis of adsorbents, micro-electro-mechanical systems, the design of functional clothing, and so on.

It is demonstrated that the gas flow in tight porous media is significantly different from that in conventional gas reservoirs. Permeability of medium-to-high permeability matrices is only a property of porous medium and irrelevant to fluid property. However, many researchers have pointed out that the permeability of tight porous media containing a large fraction of micro/nano pores or fractures is not only dependent on structural parameters of tight porous media but also on gas properties and pressure and temperature, due to the Knudsen diffusion and the compressibility of gas [1]. Up to now, the large amount of theoretical research on gas transport in porous media may be divided into two methods: numerical simulation method and analytical-theoretical method. Numerical simulation method contains the Monte Carlo simulation method, the Lattice Boltzmann method, and the molecular dynamic simulation. However, numerical simulation methods are limited by very long computing time and expensive computer memory, which make them simulate impractically gas flow in fractured porous media. Analytical-theoretical methods include slip models and volume diffusion hydrodynamics models [2]. However, most of slip models incorporate one or more empirical constants, which are very difficult to determine. Volume diffusion hydrodynamics models are based on the linear stacking of convection flow and diffusion component. In recent years, a growing number of investigators have attempted to develop theoretical models for gas transport in porous media. For the sake of clarity, a summary of available models for gas transport in porous media are illustrated in Table 1. As Table 1 indicates that some models contain the tangential momentum accommodation coefficient (TMAC) or other empirical constants, others do not consider roughness of pore surface. Moreover, literature research shows that there is no appropriate method to evaluate the value of TMAC [2,3]. 

Although a lot of gas transport models have been developed in recent years, the majority of published analytical permeability models for porous media are established based on the hypothesis about porous media consisting of smooth circular capillaries. Nevertheless, porous media in nature are composed of a great number of randomly distributed and irregular nanopores or micro/nano-fractures with diverse geometrical cross sections [9,13,14,15,16,17,18,19]. Furthermore, most of fractures/capillaries surfaces are rough. The flow part of fluid through rough-walled porous media is very complicated, and the roughness of fractures/capillaries has a significant impact on fluid transport in porous media. A few comprehensive reviews on fluid flow in rough-walled fractures were published [20,21]. In recent years, there has been an increasing interest in the impact of roughness of fractures/capillaries on transport properties in a porous medium, and interested readers can consult the relevant literature [22,23,24,25,26,27,28]. Therefore, a good understanding of the physical mechanism of fluid transport in fractured porous media with rough surfaces is essential. In addition, the literature also indicated that the effective hydraulic aperture of the rough-wall fracture is reduced due to the existence of surface roughness. Zimmerman et al. applied the lubrication theory to investigate the permeability of rough-walled rock fractures, in which the roughed fracture is regarded as sinusoidal variation wall [29]. Felisa et al. presented an analytical flow rate model for non-Newtonian fluid in a rough channel to simulate natural or artificial rock fractures [30]. Miao et al. developed a fractal permeability model for fractured rocks with smooth surfaces on the basis of the cubic law [31]. Xu et al. obtained an analytical expression for effective permeability of fractured porous media according to the fractal theory and analyzed the influence of fractal dimensions on the effective permeability [32]. However, the influence of real gas effect and fracture wall roughness on the permeability for fractured porous media was neglected in the above-mentioned articles [31,32]. Yang et al. [27] developed a permeability model based on porous media consisting of a bundle of tortuous circular capillaries with rough walls; however, they did not consider the effect of gas rarefaction on the permeability of porous media. Recently, Xiao et al. [26] presented a fractal model for Kozeny–Carman constant and dimensionless permeability of fibrous porous media by considering the influence of the roughness of capillaries surface. Wang et al. [33] proposed a new permeability model for 2D complex tortuous fractured porous media on a basis of fractal-like tree fracture network model and the cubic law but neglected the impact of surface roughness of capillaries. The concept about apparent gas permeability was initially proposed by Javadpour el at. [5] to simplify the complexity of the calculation. When pore/fracture scale is comparable to the gas molecular mean free path or the pressure is low, Knudsen diffusion is dominated. Thus, apparent gas permeability is higher than intrinsic permeability (it is also called as liquid permeability). It has been shown that gas flows in tight porous media in a low-pressure range; apparent gas permeability could vary with pressure [34]. Therefore, it is more meaningful for practical engineering application to develop an apparent gas permeability model for fractured porous media with roughened surfaces and Knudsen diffusion included. So far, few studies have investigated simultaneously the influences of real gas effect, gas rarefaction, and surface roughness of fracture on apparent gas permeability of fractured porous media. Therefore, a comprehensive and simple permeability model capturing effect of roughened surfaces, gas rarefaction, and real gas is urgently required. 

Although some researchers have studied the flow in fractal porous media with rough surfaces [22,26,27], the aim of this paper is to obtain the derivation of an analytical expression for apparent gas permeability of fractured porous media with roughened surfaces, including the effect of gas rarefaction and real gas effect based on fractal theory and technology. In the following, a brief introduction of the fractal theory and technology is first illustrated. Then in Section 3, a novel fractal model for apparent gas permeability of fractured porous media is developed based on the linear stacking of convection mass transfer and Knudsen diffusive mass transfer with consideration of fracture surface roughness and real gas effect. Next, the results and discussions of the proposed model are given in Section 4, and then comes a brief summary in Section 5.

## 2. Basic Fractal Theory

**Figure 1:**
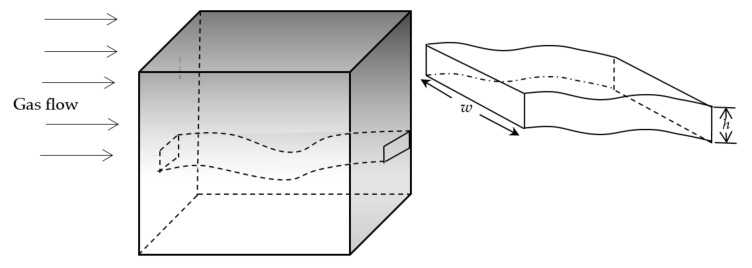
A schematic diagram of tight reservoir rocks composed of a bunch of parallel micro/nano-fractures.

Fractured porous media with roughened surfaces is ubiquitous in nature such as oil and gas reservoirs, underground water resources and nuclear waste disposal systems. In general, oil and gas reservoirs may be considered to be composed of a large number of randomly distributed fractures embedded with a low permeability porous matrix and form called a dual-porosity medium. In this work, we consider porous media which are embedded with a series of parallel rectangular section capillaries with roughened surface (i.e., micro/nano-fractures) to approximately simulate fractured porous media, as shown in Figure 1. Since the permeability of fracture networks serving as main flow pathways is much higher than that of porous matrix, fluid flow in porous matrix may be neglected in this paper. In recent years, an increasing amount of literature has presented convincing evidence that most porous media in nature are fractal objects [19,31,35,36,37,38,39]. Suppose that both aperture sizes and rough wall surface morphology of fractures satisfy the fractal power laws. The aperture and width of a single micro/nano-fractures are denoted by *h* and *w*, respectively. According to the fractal theory, the cumulative width distribution of micro/nano-fractures in tight reservoir rocks complies the following fractal scaling law [31]:

(1) $$N(W\geq w)={\left(\frac{w_{max}}{w}\right)}^{d_{f}}$$

where *d_f_* is the fractal dimension for micro/nano-fractures widths and can be calculated by $d_{f}=d_{E}-\frac{\ln\phi }{\ln\left(w_{\min}/w_{\max}\right)}$ [31], 0 < *d_f_* < 2 in two dimensions and 0 < *d_f_* < 3 in three dimensions. Owing to thousands of micro/nano-fractures in tight reservoir rocks, Equation (1) can be regarded as a continuous and differentiable function. Differentiating it with respect to *w*, one can find the number of micro/nano-fractures lying in the infinitesimal range *w*,

(2) $$dN=-d_{f}w_{max}^{d_{f}}w^{-(d_{f}+1)}dw$$

Due to the tortuous characteristic of the flow pathways, the actual length for gas transport in tight reservoir rocks is longer than the straight length *L*_0_, and the actual length $l_{t}$ can be expressed as by the tortuous fractal dimension $d_{t}$ [13],

(3) $$l_{t}=w^{1-d_{t}}L_{0}^{d_{t}}$$

Several studies have documented that the aperture of the micro/nano-fracture is proportional to its width [31,40]

(4) h=βw

where β is the proportionality coefficient.

**Figure 2:**
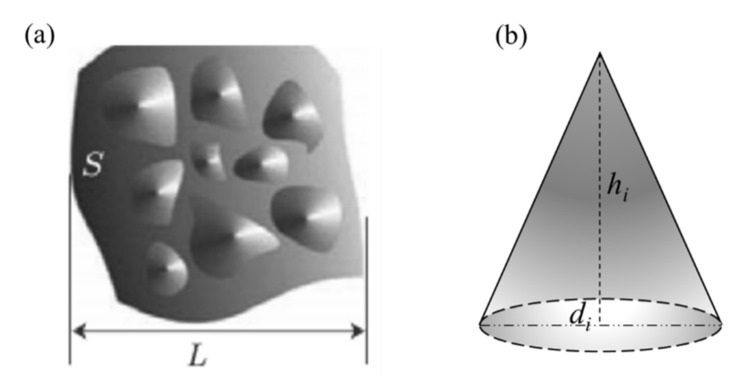
Typical morphology of a rough surface: (**a**) top view of a rough surface, (**b**) a representative cone-like rough element.

In general, wall surfaces of micro/nano-fractures in tight reservoir rocks are not smooth. Majumdar and Bhushan [41] argued the size distribution of contact spots on engineering surfaces to obey the fractal scaling law. Herwig et al. [42] carried out experimental measurements for the gas flow in micro-channels and pointed out that surface roughness played a very important role in gas microscale flows. Yang et al. [27] applied conic-shaped rough elements to characterize surface roughness of micro channels and also analyzed the effect of surface roughness on the laminar flows in micro channels. Recently, Zheng et al. [25] investigated the impact of rough morphology of pore wall of micro- and nano-porous media on gas slip flow and obtained the fractal model of gas slippage factor based on fractal theory. Furthermore, Zheng et al. [43] developed the fractal model of gas diffusion through porous fibrous materials with rough surfaces, based on the assumption of pore size distribution and surface roughness following the statistically self-similar fractal characteristics. For conic-shaped rough elements, interested readers may consult some relevant References [25,27,28,41,42,43]. In this paper, we assume that cone-shaped rough elements randomly distribute on wall surfaces of each micro/nano-fracture, as shown in Figure 2.

Generally speaking, a higher peak means a larger bottom area of a cone peak. That is to say, the ratio of the height of the peak to the base diameter of conic rough element is considered as a constant. Based on the assumption of Yang et al. [27] and Zheng et al. [25,43], the ratio of the height of the peak to the base diameter of conic rough element is considered as a constant, viz.

(5) $$\xi =h_{i}/d_{i}$$

where *h_i_* and *d_i_* denote the height and the base diameter of conic rough elements, respectively. Since the size distribution of rough elements obeys the fractal scaling law, the cumulative size distribution of rough elements on the surface wall of micro/nano-fractures is expressed as [27]

(6) $$N(l\geq d)={\left(\frac{d_{max}}{d}\right)}^{d_{r}}$$

where *N* represents the total number of conic-shaped rough elements with diameter scale *l* is larger than or equal to the base diameter *d. *$d_{\max}$ and $d_{r}$ represent the maximum base diameter and roughness fractal dimension. In Equation (6), $0<d_{r}<2$ in two dimensions and $0<d_{r}<3$ in three dimensions. Suppose that the relative roughness in micro/nano-fractures with different widths is the same value due to the self-similarity. The average height of cone-shaped rough elements ${\bar{h}}_{eff}$ is defined as the ratio of the total volume of a set of fractal cones to the total area for a unit cell. Interested readers can consult the Reference [28]. Therefore, the average height of cone-shaped rough elements in a single micro/nano-fracture with width *w* can be calculated as [27]

(7) $${\bar{h}}_{eff}=\frac{\varphi_{area}w{\left(h_{\max}\right)}_{w_{\min}}}{3w_{\min}}\frac{2-d_{r}}{3-d_{r}}\frac{1-{\left(\frac{w_{\min}}{w_{\max}}\right)}^{3-d_{r}}}{1-\varphi_{area}}$$

where $\varphi_{area}$ is the area ratio of the total base area of the whole conic rough elements to the cross-sectional area of a unit area. $w_{\min}$ denotes the minimum width of microfracture, and ${\left(h_{\max}\right)}_{w_{\min}}$ represents the maximum height of the rough element in the fracture with the minimum width $w_{\min}$. According to the definition of the relative roughness, the relative roughness in a single micro/nano-fracture with aperture *h* is expressed as

(8) $$\varepsilon =\frac{2{\bar{h}}_{eff}}{h}$$

Then, the actual aperture of a single micro/nano-fracture with aperture *h* is obtained as

(9) $$h_{r}=h-2{\bar{h}}_{eff}$$

The convection mass flow rate through a single tortuous micro/nano-fracture with smooth surface is obtained by soluting the Navier–Stokes equations with no-slip boundary condition [6]

(10) $$m_{c}=\frac{wh^{3}Mp\Delta p}{12\mu RZTl_{t}}$$

Considering the rough surface of microfractures in tight reservoir rocks, the aperture of micro/nano-fracture will be reduced. Therefore, Equation (10) will be modified as

(11) $$m_{c}=\frac{w{\left(h-2{\bar{h}}_{eff}\right)}^{3}Mp\Delta p}{12\mu RZTl_{t}}$$

where *Z* is the gas dimensionless compressibility factor, and *R* and *M* are the universal gas constant (Pa · m^3^/(mol· K)), and the gas molar mass (g/mol), respectively. The gas viscosity μ depends on the temperature, pressure, and gas properties, which are given by Lee et al. [44],

(12) $$\mu =c\exp(X\rho^{Y})$$

(13) $$c=\frac{(9.4+0.02M)T^{1.5}}{209+19M+T}$$

(14) $$X=3.5+\frac{986}{T}+0.01M$$

(15) Y=2.4−0.2X

where ρ and *M* are gas density and gas molecular molar mass, respectively.

Inserting Equations (3) and (8) into Equation (11), Equation (11) can be rewritten as

(16) $$m_{c}=\frac{\beta^{3}w^{3+d_{t}}{\left(1-\varepsilon \right)}^{3}Mp\Delta p}{12\mu RZTL_{0}^{d_{t}}}$$

Due to the fact that numerous microscale and nanoscale microfractures coexist in tight reservoir rocks, Knudsen diffusion occurs. The Knudsen diffusion mass flow rate in the single micro/nano-fracture with smooth surface is obtained by Fick’s first law, which is expressed as [13]

(17) $$m_{k}=\frac{8h^{2}w\Delta p}{3\pi l_{t}}\sqrt{\frac{2M}{\pi ZRT}}$$

However, the surface of micro/nano-fracture is seldom smooth, the aperture of micro/nano-fracture will be reduced by considering the surface roughness of micro/nano-fractures. Then Knudsen diffusion mass flow rate in the single micro/nano-fracture with rough surface can be corrected as

(18) $$m_{k}=\frac{8{\left(h-2{\bar{h}}_{eff}\right)}^{2}w\Delta p}{3\pi l_{t}}\sqrt{\frac{2M}{\pi ZRT}}$$

With the aid of Equations (3) and (8), Equation (18) can be rewritten as

(19) $$m_{k}=\frac{8\beta^{2}w^{2+d_{t}}{\left(1-\varepsilon \right)}^{2}\Delta p}{3\pi L_{0}^{d_{t}}}\sqrt{\frac{2M}{\pi ZRT}}$$

Z-Factor is a key thermodynamic parameter in the petroleum and chemical engineering disciplines [45], which is expressed as

(20) $$Z=1+(A_{1}+\frac{A_{2}}{T_{r}}+\frac{A_{3}}{T_{r}^{3}}+\frac{A_{4}}{T_{r}^{4}}+\frac{A_{5}}{T_{r}^{5}})\rho_{r}+(A_{6}+\frac{A_{7}}{T_{r}}+\frac{A_{8}}{T_{r}^{2}})\rho_{r}^{2}-A_{9}(\frac{A_{7}}{T_{r}}+\frac{A_{8}}{T_{r}^{2}})\rho_{r}^{2}\;+[\frac{A_{10}}{T_{r}^{3}}\rho_{r}^{2}(1+A_{11}\rho_{r}^{2})exp(-A_{11}\rho_{r}^{2})]$$

where $A_{1}=0.3265$, $A_{2}=-1.0700$, $A_{3}=-0.5339$, $A_{4}=0.01569$, $A_{5}=-0.05165$, $A_{6}=0.5475$, $A_{7}=-0.736$, $A_{8}=0.1844$, $A_{9}=0.1056$, $A_{10}=0.6314$, $A_{11}=0.721$.

The reduced density can be expressed as [45]

(21) $$\rho_{r}=\frac{0.27p_{r}}{ZT_{r}}$$

where $T_{r}$ and $p_{r}$ are the reduced temperature and the reduced pressure, respectively. Once the values of $T_{r}$ and $p_{r}$ are given, the value of Z-Factor can be calculated by Newton–Raphson iterative algorithm.

## 3. A Novel Fractal Model for Gas Apparent Permeability of Tight Reservoir Rocks with Rough Surfaces

The mechanism of gas transport in tight/shale reservoirs is very complicated. Convection volume flow and Knudsen diffusion coexist in shale porous media. It is noted that surface diffusion is beyond the scope of this paper, which is our future study. For the sake of simplicity, we assume that convection volume flow and Knudsen diffusion do not interact with each other. However, convection volume flow and Knudsen diffusion may interact in a more complicated manner in nature, and this is still an open question and will be the subject of our next study. Assuming the slip flow can be regarded as a part of Knudsen diffusion [2], the volumetric flow rate through a single fracture is taken as the linear superposition of convection volumetric flow rate and Knudsen diffusive volumetric flow rate as follows:

(22) $$m(w)=m_{c}+m_{k}$$

In this section, we focus our attention on developing a fractal model for apparent gas permeability of tight reservoir rocks with the consideration of surface roughness of micro/nano-fracture walls, based on the superposition of convection transfer and Knudsen diffusion. The total mass flow through all the micro/nano-fractures in tight reservoir rocks can be calculated by integrating Equation (22) from the minimum width to the maximum width in a unit cell, i.e.,

(23) $$m_{total}=-\int_{w_{\min}}^{w_{\max}}(m_{c}(w)+m_{k}(w))dN\;=\frac{{\left(1-\varepsilon \right)}^{3}\beta^{3}d_{f}Mp_{m}w_{\max}^{3+d_{t}}}{12\mu ZRTL_{0}^{d_{t}-1}(3-d_{f}+d_{t})}\frac{\Delta p}{L_{0}}[1-{\left(\frac{w_{\min}}{w_{\max}}\right)}^{3-d_{f}+d_{t}}]\;+\frac{8\beta^{2}{\left(1-\varepsilon \right)}^{2}d_{f}w_{\max}^{2+d_{t}}}{3\pi L_{0}^{d_{t}-1}(2-d_{f}+d_{t})}\sqrt{\frac{2M}{\pi ZRT}}\frac{\Delta p}{L_{0}}[1-{\left(\frac{w_{\min}}{w_{\max}}\right)}^{2-d_{f}+d_{t}}]$$

According to the general Darcy’s law, we can get the apparent gas permeability expression for gas flow through tight reservoir rocks composed of micro/nano-fractures as follows:

(24) $$k_{as}=\frac{\mu m_{total}}{\rho A\Delta p/L_{0}}\;=\frac{{\left(1-\varepsilon \right)}^{3}\beta^{3}d_{f}w_{\max}^{3+d_{t}}}{12L_{0}^{d_{t}-1}(3-d_{f}+d_{t})A}[1-{\left(\frac{w_{\min}}{w_{\max}}\right)}^{3-d_{f}+d_{t}}]\;+\frac{8\mu \beta^{2}{\left(1-\varepsilon \right)}^{2}d_{f}w_{\max}^{2+d_{t}}}{3\pi pL_{0}^{d_{t}-1}A(2-d_{f}+d_{t})}\sqrt{\frac{2ZRT}{\pi M}}[1-{\left(\frac{w_{\min}}{w_{\max}}\right)}^{2-d_{f}+d_{t}}]$$

It is noted that the area *A* in Equation (24) represents the total cross-sectional area of a unit cell which contains a set of fractal fractures, instead of the selected sample area, and the gas density ρ is expressed as $\rho =\frac{pM}{ZRT}$. Due to $1<d_{t}<3$, $1<d_{f}<3$, and $\frac{w_{\min}}{w_{\max}}\approx 10^{-2}$, ${\left(\frac{w_{\min}}{w_{\max}}\right)}^{3-d_{f}+d_{t}}<<1$ ${\left(\frac{w_{\min}}{w_{\max}}\right)}^{2-d_{f}+d_{t}}<<1$ in porous media, Equation (24) can be simplified into

(25) $$k_{as}=\frac{{\left(1-\varepsilon \right)}^{3}\beta^{3}d_{f}w_{\max}^{3+d_{t}}}{12L_{0}^{d_{t}-1}(3-d_{f}+d_{t})A}\;+\frac{8\mu \beta^{2}{\left(1-\varepsilon \right)}^{2}d_{f}w_{\max}^{2+d_{t}}}{3\pi pL_{0}^{d_{t}-1}(2-d_{f}+d_{t})A}\sqrt{\frac{2ZRT}{\pi M}}$$

Equation (25) is the fractal model for apparent gas permeability in fractured porous media with rough micro/nano-fractures. From Equation (25), it is evident that gas apparent permeability is expressed as a function of relative roughness, micro-structural parameters of tight reservoir rocks, gas properties, pressure, and temperature and is free of empirical constants. The present fractal apparent gas permeability model can reveal the physical mechanism that affects apparent gas permeability in fractured porous media.

If the micro/nano-fracture walls are smooth, i.e., ε=0, Equation (25) can be reduced to be

(26) $$k_{as}=\frac{\beta^{3}d_{f}w_{\max}^{3+d_{t}}}{12L_{0}^{d_{t}-1}(3-d_{f}+d_{t})A}\;+\frac{8\mu \beta^{2}d_{f}w_{\max}^{2+d_{t}}}{3\pi pL_{0}^{d_{t}-1}(2-d_{f}+d_{t})A}\sqrt{\frac{2ZRT}{\pi M}}$$

Equation (26) is exactly the apparent gas permeability model for gas transfer in tight porous media with smooth micro/nano-fractures and coincides with the result of Wang et al. model [13], whose model is valid only for tight porous media consisting of smooth micro/nano-fractures and ideal gas. Therefore, our present apparent gas permeability model Equation (25) can be regarded as an extension of Wang et al. model, which is suitable for tight porous media composed of micro/nano-fractures including rough surfaces of fractures and real gas effect.

## 4. The Result and Discussion

In this section, the proposed apparent gas permeability model for gas flow in tight reservoir rocks composed of micro/nano-fractures with rough surfaces is compared with the existing model and experimental data collected from available literature. The total cross-sectional area *A* of the unit cell can be expressed as [13,38,46,47]

(27) $$\begin{array}{ll} A=\frac{A_{p}}{\phi } & =\frac{-\int_{w_{\min}}^{w_{\max}}w\cdot (h-2{\bar{h}}_{eff})\cdot dN}{\phi }\\ & =\frac{\beta (1-\varepsilon )d_{f}w_{\max}^{2}}{2-d_{f}}\frac{1-\phi }{\phi } \end{array}$$

If the tight reservoir rock can be approximated as square cross section, the straight length along gas transfer direction is given by [13,38,46,47]

(28) $$L_{0}=\sqrt{A}$$

Inserting Equations (27) and (28) into Equation (25), we can obtain the apparent gas permeability

(29) $$k_{as}=\frac{{\left(1-\varepsilon \right)}^{3}\beta^{3}d_{f}w_{\max}^{3+d_{t}}}{12(3-d_{f}+d_{t})}\times {\left[\frac{\phi (2-d_{f})}{\beta (1-\varepsilon )d_{f}w_{\max}^{2}(1-\phi )}\right]}^{\frac{d_{t}+1}{2}}\;+\frac{8\mu \beta^{2}{\left(1-\varepsilon \right)}^{2}d_{f}w_{\max}^{2+d_{t}}}{3\pi p(2-d_{f}+d_{t})}\sqrt{\frac{2ZRT}{\pi M}}\times {\left[\frac{\phi (2-d_{f})}{\beta (1-\varepsilon )d_{f}w_{\max}^{2}(1-\phi )}\right]}^{\frac{d_{t}+1}{2}}$$

Thus, the structural parameters of the unit cell (viz. the total cross sectional area *A* of a unit cell, representative length of *L*_0_) do not occur in the above apparent gas permeability expression Equation (29) anymore. Therefore, the apparent gas permeability of fractured porous media with rough micro/nano-fractures only depends on the relative roughness, micro-structural parameters of tight reservoir rocks ($\beta ,d_{f},d_{t}w_{\max},\phi$), gas properties, pressure, and temperature and is free of empirical constants. Each parameter has a clear physical meaning in the above apparent gas permeability expression Equation (29), which can reflect the physical mechanism of the apparent gas permeability of fractured porous media with rough micro/nano-fractures.

Singh et al. [8] proposed an analytical expression for apparent gas permeability of a porous medium consisting of slits with the tortuosity of flow path and the real gas effect included as follows:

(30) $${\left(k_{a}\right)}_{slit}=\frac{\phi }{\tau }\frac{\mu h}{3}(\frac{hZ}{4\mu }+\frac{8}{\pi pM}\sqrt{\frac{2MRT}{\pi }})$$

The aforementioned permeability model Equation (30) is free of any empirical coefficients and depends on microstructural parameters (such as the aperture, porosity, and tortuosity), temperature, average reservoir pressure, and gas properties. However, Singh et al. did not consider the effect of wall roughness of slits and aperture size distribution in his model.

The fractal dimension for $d_{f}$ in Equation (25) can be calculated from $d_{f}=d_{E}-\frac{\ln\phi }{\ln(w_{\min}/w_{\max})}$ [31]. It is noted that the tortuosity in Singh et al. model can be determined by $\tau =1+0.63\ln\frac{1}{\phi }$ [48].

**Figure 3:**
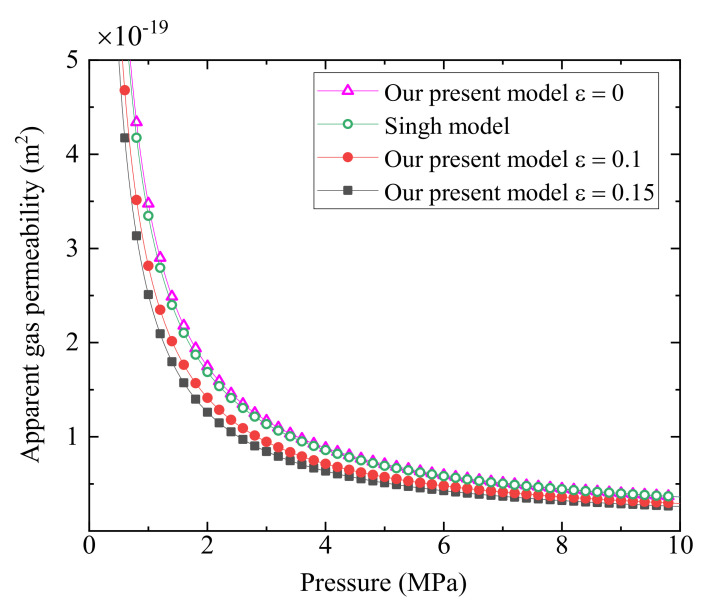
A comparison of apparent gas permeability between the present model Equation (25) and the available model [8].

Figure 3 shows a comparison of existing model with the predicted values based on fractal apparent permeability model given by Equation (25) with the parameter $d_{t}=1.1$ (the same value as that in available references [38,49]), and other parameters are illustrated on Table 2. As we can observe from Figure 3, our apparent gas permeability model (Equation (25)) for fractured porous media based on the fractal theory at the relative roughness ε=0 matches well with Singh et al. model [8]. However, Singh et al. model works for tight porous media composed of with micro/nano-fractures with smooth surfaces and neglects aperture size distribution. Taking a close look at Figure 3, we also observe that the predictions from our present model by Equation (25) with smooth surfaces are slightly higher than those by the model with rough surfaces at the relative roughness ε=0.1 and ε=0.15, which is in conformity with practical situation. This is because larger relative roughness results in smaller fracture aperture and larger flow resistance, causing the lower apparent gas permeability. This implies that the surface roughness has a critical influence on the apparent gas permeability.

**Figure 4:**
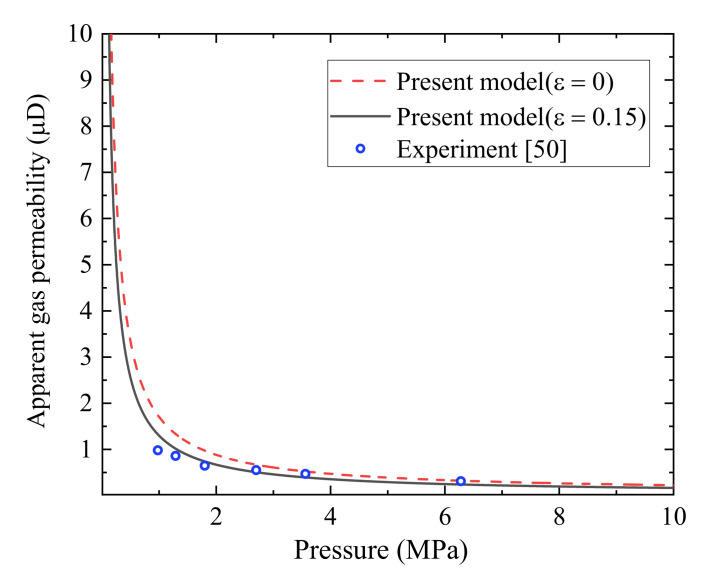
A comparison on gas apparent permeability of tight reservoir rocks between our fractal analytical model and existing experimental data [50].

Figure 4 represents a comparison between the predictions from the present fractal apparent permeability under different rough surfaces based on Equation (25) and experimental data [50]. Letham [50] measured the permeability for helium gas through a green-grey parallel laminated siltstone from the Horseshoe Canyon Formation over the range of sample pressure from 1 to 8 MPa by the pressure pulse decay technique. Since the microstructural parameters of shale sample are not available in the original work, we can give reasonable values to match experimental data well. In addition, the maximum width and the minimum one are assumed reasonably to be 500 and 5nm, respectively. The porosity of shale sample is lower than 0.1, so the value of porosity of shale sample is set as 0.04. As shown in Figure 4, a fair agreement between the predictions from the present apparent gas permeability model at ε=0.15 and the experimental data is obtained. The validity of fractal apparent permeability model for real gas flow in fractured porous media with roughened surfaces is tested.

**Figure 5:**
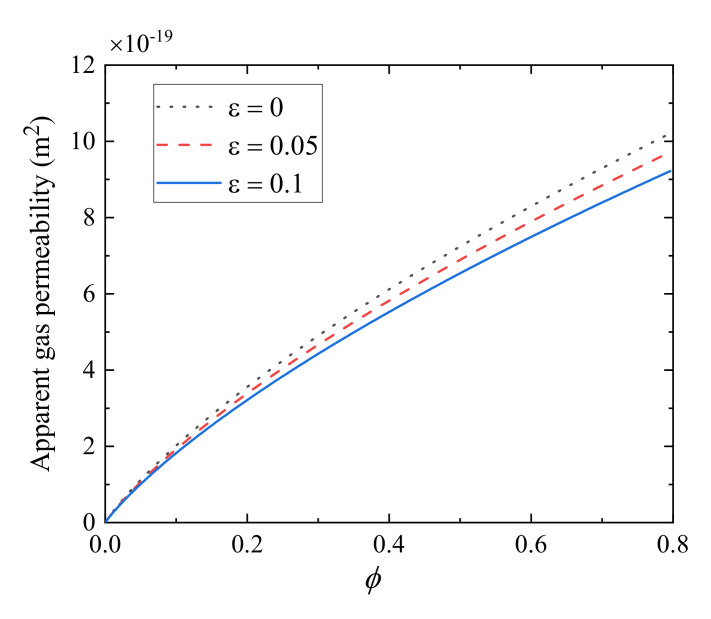
Effect of the porosity on the apparent permeability at different relative roughness.

**Figure 6:**
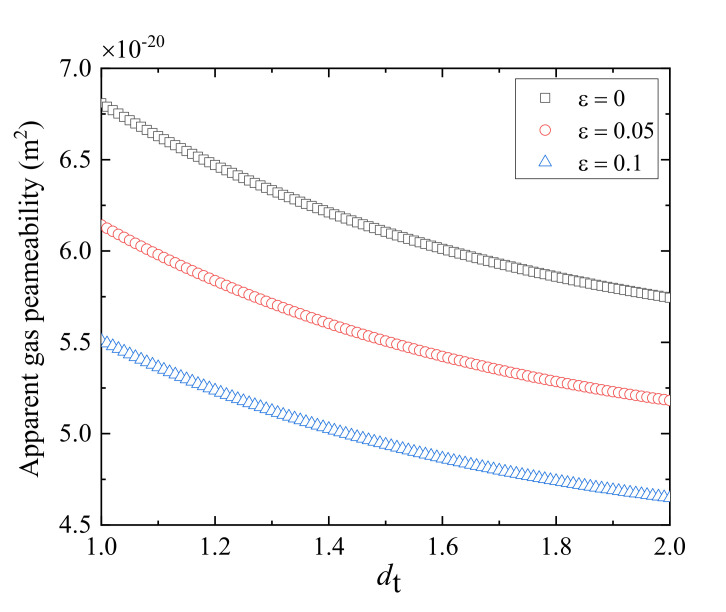
The impact of tortuosity fractal dimension on the apparent gas permeability at different relative roughness.

**Figure 7:**
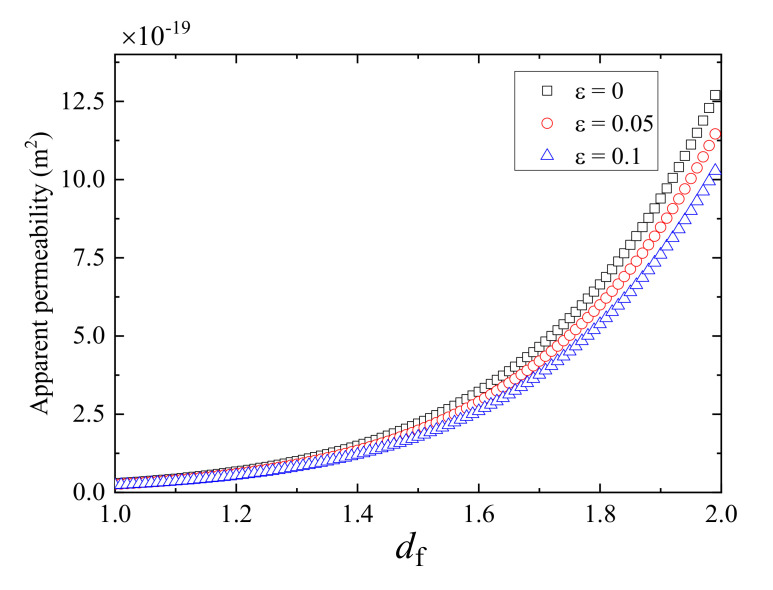
Plot of apparent gas permeability against the area fractal dimension at different relative roughness.

**Figure 8:**
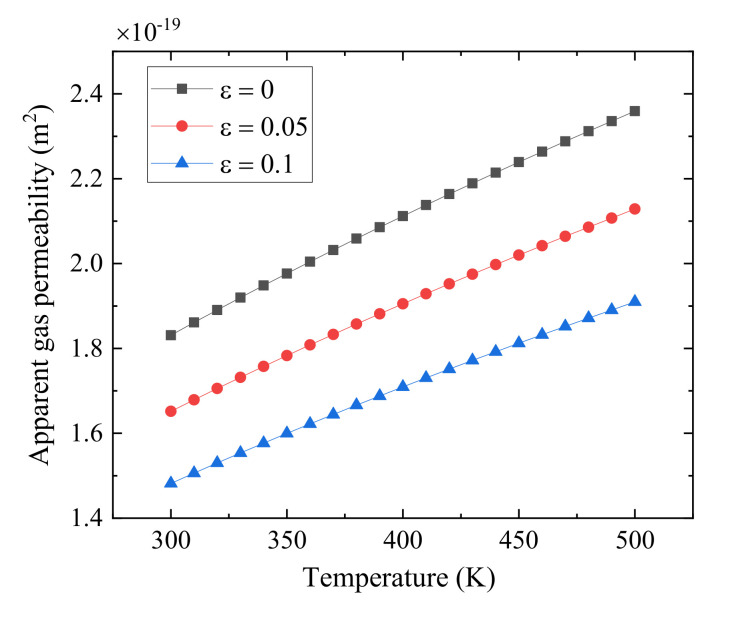
Plot of apparent permeability against temperature at different relative roughness.

Figure 5 plots helium gas apparent permeability, *k*_as_, versus the porosity at different relative roughness at the given mean pressure *p* = 2 MPa and temperature *T* = 300 K. As illustrated in Figure 5, the apparent gas permeability increases monotonously with the increase of the porosity. This can explain that larger porosity corresponds to larger fracture space, leading to more easy gas transport in tight porous media and higher apparent gas permeability. It can also be seen from Figure 5 that relative roughness has an impact on apparent gas permeability, i.e., the larger relative roughness, the lower apparent gas permeability. As we mentioned previously, the larger relative roughness means the smaller fracture aperture, resulting in a lower apparent gas permeability. Similar phenomena can be also observed in Figure 3, Figure 4, Figure 5, Figure 6, Figure 7 and Figure 8.

Figure 6 demonstrates the variation of apparent gas permeability for helium gas in fractured porous media against the tortuosity fractal dimension. It is found that the apparent gas permeability decreases markedly with the increase of the tortuosity fractal dimension. This may be attributed to the fact that the larger tortuosity fractal dimension, the higher flow resistance, leading to a lower apparent gas permeability.

Figure 7 depicts the effect of area fractal dimension, *d*_f_, on the apparent gas permeability. It can be seen that the apparent gas permeability increases significantly with the increasing fractal dimension. This can be interpreted as that the larger the area fractal dimension means the larger porosity based on the correlation *d*_f_ = d_E_ − ln*ɸ*/ln(*w*_min_/*w*_max_) [31], which results in larger pore space for gas flow in fractured porous media and lower flow resistance, leading to higher apparent gas permeability for gas transport in fractured porous media.

Figure 8 shows the impact of the temperature on the apparent gas permeability at the different relative roughness. It can be observed that the gas apparent permeability increases significantly with the increase of the temperature at the fixed relative roughness. This is because the higher temperature, the larger Knudsen number, leading the more remarkable Knudsen diffusion and higher apparent permeability from Knudsen diffusion. Therefore, the apparent gas permeability is increased with the increase of temperature based on Equation (25).

## 5. Conclusions

A novel apparent gas permeability model has been developed in fractured porous media consisting of micro/nano-fractures with rough rectangular cross-sections, which is based on fractal theory and technology and the assumption of the linear superposition of convection transfer and Knudsen diffusive transfer. The proposed apparent gas permeability model is in terms of relative roughness, micro-structural parameters of fractured porous media, gas properties, pressure, temperature, which contains no empirical constants. Each parameter has a clear physical meaning in our present apparent gas permeability model, which can reflect the physical mechanism of the apparent gas permeability of fractured porous media with rough micro/nano-fractures. The present gas permeability model is confirmed by comparing its predictions with the existing experimental data and the available model in the literature. Results indicate that our present model agrees well with the existing experimental data and the available model, which verifies the reasonability of our present model. In this paper, we mainly focus on the fluid flow characteristics in fractures with roughened surfaces and neglect the seepage flow characteristics of porous matrix. In reality, real fracture networks in nature are extremely complex, and some of them may be connected and overlap one another. For this case, a more comprehensive and complicated permeability model for fractured porous media is required to be developed. This paper just carries through the preliminary research, and there is much extended work to do. In addition, it is also a challenging task to numerically simulate a real 3-D fracture networks, due to the limitations of computer memory and computation time. The proposed model can provide better understanding of transport mechanisms of gas flow in fractured porous media. However, many other factors, such as the type of fracture, stress sensitivity, and surface diffusion, may affect apparent gas permeability. A more realistic model than the present one may need to be developed in our future work.

## Figures and Tables

**Table 1:** A summary of different gas transport models.

Model	Description	Comment
Beskok and Karniadakis (1999) [4]	A unified Hagen–Poiseuille type equation to describe various flow regimes	two empirical constants introduced, suitable for a pipe and a rectangular channel with smooth surfaces
Javadpour (2009) [5]	A model for fluid flow in a single, straight, and cylindrical nanochannel with Knudsen diffusion and slip flow included	TMAC introduced, only suitable for a tube with smooth surfaces
Thomas Veltzke and Jorg Thöming (2012) [6]	A model developed based on superposition of convective transport and Fickian diffusion	without TMAC and suitable for a tube and a rectangular channel with smooth surfaces
Darabi et al. (2012) [1]	The extension of Javadpour model	TMAC introduced; only suitable for circular tube and smooth walls
Ziarani and Aguilera (2012) [7]	Knudsen’s Permeability model developed based on Beskok and Karniadakis model for microflows	some empirical constants introduced
Singh and Javadpour (2014) [8]	A model developed based on superposition of convective transport and Knudsen diffusion	suitable for a tube and a rectangular channel; without the effect of rough surface morphology on gas transport
Wu et al. (2015) [9]	A model developed based on the weighted superposition of slip flow and Knudsen diffusion	slip effect, real gas effect, the impact of nanopore type and shape included; without the consideration of pore wall roughness
Wu et al. (2015) [10]	A model developed by coupling slip flow and Knudsen diffusion together using the weighted coefficients	weighted factor included; suitable for fractures with rectangular cross-sections with smooth surfaces
Yuan et al. (2016) [11]	A model developed based on Beskok and Karniadakis general slip boundary condition and fractal theory	suitable for circular capillaries with smooth surfaces
Cai et al. (2020) [12]	A fractal permeability model developed with consideration of axial stress and creeping microstructure	suitable for circular capillaries with smooth surfaces

**Table 2:** Model structural parameters and gas properties.

Parameter	Value	Description
$w_{\min}$	1 nm	the minimum aperture of tight reservoir rocks
$w_{\max}$	100 nm [13]	the maximum aperture of tight reservoir rocks
β	0.01 [13]	the proportionality coefficient
ϕ	0.08	the porosity
*M*	4 g/mol	the Helium molar mass
*T*	300 K	Temperature

## Data Availability

The data presented in this study are available on request from the corresponding author.
